# *Helicobacter pylori* CagA promotes epithelial mesenchymal transition in gastric carcinogenesis via triggering oncogenic YAP pathway

**DOI:** 10.1186/s13046-018-0962-5

**Published:** 2018-11-22

**Authors:** Nianshuang Li, Yan Feng, Yi Hu, Cong He, Chuan Xie, Yaobin Ouyang, Stephen C. Artim, Deqiang Huang, Yin Zhu, Zhijun Luo, Zhongming Ge, Nonghua Lu

**Affiliations:** 1 Department of Gastroenterology, The First Affiliated Hospital of Nanchang University, 17 Yong Waizheng Street, Donghu District, Nanchang, 330006 Jiangxi Province China; 2 Division of Comparative Medicine, Massachusetts Institute of Technology, 77 Massachusetts Avenue, Cambridge, MA 02139 USA; 3 Department of Biochemistry, Boston University School of Medicine, 72 East Concord Street, Boston, MA 02118 USA

**Keywords:** *H. pylori* CagA, YAP, Epithelial-mesenchymal transition, Gastric carcinogenesis

## Abstract

**Background:**

*Helicobacter pylori* (*H. pylori*) delivers oncoprotein CagA into gastric epithelial cells via the T4SS and drives activation of multiple oncogenic signalling pathways. YAP, a core effector of the Hippo tumour suppressor pathway, is frequently overexpressed in human cancers, suggesting its potential tumor-promoting role. Although CagA is a casual factor in *H. pylori* induced gastric carcinogenesis, the link between CagA and YAP pathway has not been identified. In this work, we investigated the regulation of oncogenic YAP pathway by *H. pylori* CagA.

**Methods:**

Expression of YAP and E-cadherin protein in human gastric biopsies were assessed by immunohistochemistry. *H. pylori *PMSS1 *cagA*^*−*^ isogenic mutant strains were generated. Gastric epithelial cells were co-cultured with *H. pylori* wild-type *cagA*^*+*^ strains or isogenic mutants and were also treated by recombinant CagA expression. Immunofluorescence was performed for YAP localization. Immunoblot and quantitative PCR were performed for examining levels of YAP, downstream effectors and markers of epithelial-mesenchymal transition. Verteporfin and siRNA silencing were used to inhibit YAP activity.

**Results:**

YAP is significantly upregulated in human gastric carcinogenesis. We generated PMSS1 CagA isogenic mutant strains with chloramphenicol resistance successfully. Our analysis indicated that *H. pylori* infection induced YAP and downstream effectors in gastric epithelial cells. Importantly, knockout of CagA in 7.13 and PMSS1 strains reduced the expression of YAP by *H. pylori* infection. Moreover, Inhibition of YAP suppressed *H. pylori* infection-induced Epithelial-mesenchymal transition (EMT).

**Conclusion:**

Our results indicated that *H. pylori* CagA as a pathogenic protein promotes oncogenic YAP pathway, which contributes to EMT and gastric tumorigenesis. This study provided a novel mechanistic insight into why *cagA*^*+*^ *H. pylori* infection is associated with a higher risk for the development of gastric cancer.

**Electronic supplementary material:**

The online version of this article (10.1186/s13046-018-0962-5) contains supplementary material, which is available to authorized users.

## Introduction

The gram-negative microaerophilic bacterium, *Helicobacter pylori* (*H. pylori*), infects approximately half of the world’s population and is mostly acquired in childhood [1, 2]. It has been estimated that 80–90% of *H. pylori* infection is asymptomatic; 10–15% and 1–3% of *H. pylori*-infected individuals develop gastric ulcer and gastric adenocarcinoma respectively [3]. *H. pylori* infection can lead to gastric carcinogenesis through the histopathological Correa cascade of steps which include atrophic chronic gastritis, intestinal metaplasia and dysplasia culminating in gastric cancer [4]. The clinical outcome of *H. pylori* infection is determined by multiple factors including pathogenicity of individual *H. pylori* strains, host susceptibility and environmental stimuli [5, 6]. Epidemiological data indicate that infection with *cagA+* (cytotoxin-associated gene A) *H. pylori* strains is associated with more severe gastric inflammation and a higher risk for the development of pre-neoplastic lesions including intestinal metaplasia and dysplasia in comparison with *cagA- H. pylori* strains [7]. It has been also documented that CagA plays an important role in *H. pylori*-induced gastric tumorigenesis in rodent models [8, 9]. CagA is encoded on the *cag* pathogenicity island and can be delivered into gastric epithelial cells through Type IV secretion system [10]. Upon delivery into the target cells, CagA promotes various pro-oncogenic signalling pathways [11]. Notably, CagA in the host cells is tyrosine-phosphorylated and interacts with SHP-2, leading to cellular morphological changes associated with increased cell motility and scattering, termed the “hummingbird phenotype” [12, 13].

The mammalian Hippo tumor suppressor signalling pathway is crucial in maintaining developmental organ size and tissue homeostasis [14]. The central components of the Hippo pathway comprise MOB1 (Mps One Binder kinase activator), Sav1 (also known as WW45), MST1/2 (STE20-like protein kinase 1), LATS1/2 (large tumor suppressor 1) and two major downstream effectors YAP (Yes-Associated Protein), and transcriptional co-activator TAZ (PDZ-binding motif) [15]. Canonically, when this signalling is on, MST1/2 phosphorylates LATS1/2 at Thr1079/Thr1041 sites, stimulated by SAV1 and MOB1. Then phosphorylated LATS1 directly phosphorylates YAP at Ser127, resulting in cytoplasmic sequestration via binding to 14–3-3 proteins. By contrast, when Hippo signalling is off, YAP is activated and translocated from cytoplasm into nucleus through interaction with transcriptional factors TEADs [16, 17]. This process leads to expression of downstream oncoproteins, such as connective tissue growth factor (*CTGF*), cysteine-rich angiogenic inducer 61 (*CYR61*) and *MYC*. Increased expression of YAP is positively associated with progression of different human cancers [18]. Inducible overexpression of YAP in mouse liver can lead to expansion of liver size and eventual hepatocellular carcinoma [19]. Activation of YAP is functionally important for proliferative and pro-survival activity in colon cancer cell lines [20]. Therefore, YAP is considered an oncogenic protein.

Epithelial mesenchymal transition (EMT), a hallmark of tumorigenic transformation, is a cellular program by which epithelial cells lose cell-cell adhesion and acquire mesenchymal traits [21][. During this process, epithelial cells undergo marked biochemical changes, including enhanced cell elongation, loss of polarity, and migratory capacity. Expression of some common biomarkers involving cellular proliferation, including suppression of epithelial markers (e.g. E-cadherin, ZO-1) and upregulation of mesenchymal markers (e.g. N-cadherin, Vimentin, Snail and Slug), can promote EMT [22]. It has been experimentally documented that overexpression of YAP results in reduction of the epithelial marker E-cadherin and phenotypic alteration that is associated with EMT, promoting cancer cell invasion and metastasis [23, 24].

Additionally, it has been reported that intracellular CagA can disrupt cell-cell junctions and cause loss of epithelial adhesion, thereby directly activating EMT [25]. Moreover, a recent study showed that *YAP* mRNA levels were significantly elevated by *H. pylori* SS1 infection in C57BL/6 mice in combination with administration of 1-methyl-3-nitro-1-nitrosoguanidine (MNNG) [26]. However, the role of CagA in *H. pylori*-induced activation of YAP signalling pathway is poorly understood. In this study, by using CagA^+^ *H. pylori* strains PMSS1 and 7.13 as well as their *ΔcagA* mutants, we characterized effects of CagA on activation of the YAP signalling pathway in AGS cells. Also, we examined how *H. pylori* infection in humans influenced expression of YAP and an epithelial marker E-cadherin during progression of a cascade of gastric cancer from chronic non-atrophic gastritis (CNAG), intestinal metaplasia (IM), dysplasia (Dys) to cancer (GC).

## Materials and methods

### Antibodies, siRNA and plasmids

Antibodies and their sources were as follows: YAP inhibitor verteporfin from Sigma-Aldrich (St. Louis, MO, USA) for western blot assay, Anti-GAPDH (#2118), Anti-YAP (#4912), Anti-Phospho-YAP Ser127 (#4911), and Anti-Slug (#9585), from Cell Signaling Technology (Beverly, MA, USA); Anti-TAZ (HPA007415) from Sigma (St. Louis, MO, USA) (Anti-E-cadherin (#610405), Anti-N-cadherin (#610921) from BD Biosciences (San Jose, CA USA); Anti-CagA (sc-28,368) and Anti-phospho-tyrosine (sc-7020) from Santa Cruz Biotechnology (Santa Cruz, CA, USA); anti-*H. pylori* urease B (ab127916) from Abcam (Cambridge, MA, USA). For immunohistochemistry assay, Anti-YAP (#4912) from Cell Signaling Technology, Anti-TAZ (HPA007415) from Sigma, Anti-E-cadherin (#610405) from BD Biosciences For immunofluorescence assay, Anti-YAP (#4912) from Cell Signaling Technology, 4′,6-diamidino-2-phenylindole (DAPI), fluorescein isothiocyanate (FITC)-conjugated donkey anti-rabbit antibodies from Invitrogen (Thermo Fisher Scientific, Suwanee, GA, USA). The recombinant plasmid of YAP CDNA, CagA were constructed and purchased from GeneChem, Shanghai, China. YAP siRNA was purchase from Santa Cruz Biotechnology.

### *H. pylori* strains and generation of PMSS1 Δ*cagA* isogenic mutants

CagA^+^ *H. pylori* strain PMSS1 (pre-mouse Sydney strain 1) and its mouse-adapted SS1 which is deficient in CagA function due to a mutation in *cagY* were used in this study. *H. pylori* strain 7.13 and an isogenic *cagA* mutant were also included in this study, which were kindly provided by Dr. Richard Peek at Vanderbilt University Medical Center, Nashville, TN, USA. All *H. pylori* strains were cultured on trypticase soy agar with 5% sheep blood agar plates (Thermo Fisher Scientific) for in vitro passage.

For constructing PMSS1 Δ*cagA* isogenic mutants, an overlapping PCR amplicon (namely cagAdel) consisting of the upstream and downstream regions of the PMSS1 *cagA* gene(s) were produced using a pair of primers *cagA*upF and *cagA*L-5’RXS or *cagA*dnR and *cagA*F-3’FXS respectively as described previously (Additional file 3: Fig. S3A). Subsequently, a 0.7-kb *cat* (chloramphenicol acetyltransferease) cassette was ligated into a SmaI site in the overlapping site of cagAdel; two recombinant plasmids 54 and 55 which had the opposite orientations in reference to that of *cagA* were selected (Additional file 3: Fig. S3B and 4C). Then these recombinant plasmids were introduced into PMSS1 strain by electroporation or natural transformation as described previously [27]. Transformants were screened on sheep blood agar containing 25 μg/ml of chloramphenicol; four Cm^R^ transformants, namely 54E, 54 N, 55E and 55 N, were obtained. Complete deletion of *cagA* from the genomes of these transformants were verified using PCR with four primer sets F1/R1 and F2/R2 for targeting *cagA*, and Fup/54 and Fup/55 for detecting the locus containing the sequence upstream of *cagA* and the *cat* cassette as well as direct sequencing. The sequences of all primers were listed in Additional file 6: Table S3.

### Cell culture and cell transfection

Human gastric epithelial cells AGS and MKN-45 (CRL-1739; ATCC, Manassas, VA, USA) were cultured in RPMI 1640 (Gibco, CA, USA) containing 10% fetal bovine serum (Sigma Aldrich, MO, USA) and 1% penicillin/streptomycin (Gibco) at 37 °C in 5% CO_2_ atmosphere. AGS cells were transiently transfected with the YAP CDNA plasmid using Lipofectamine 2000 (Invitrogen) according to the manufacturer’s instructions.

When the cells reached 70% confluence, they were serum-starved overnight prior to *H. pylori* infection. All *H. pylori* strains were grown in Brucella broth supplemented with 5% fetal bovine serum at 37 °C for 24 h under microaerobic conditions; OD_600nm_ of bacterial suspensions was then adjusted with 1% FBS DMEM media to concentrations corresponding to a multiplicity of infection (MOI; the number of bacteria per cell at the onset of infection) of 50, 100 and 200. After co-culture with *H. pylori* strains for 6 or 24 h, AGS cells were collected for qPCR, Western blotting, or immunofluorescent staining.

### Western blotting

Cells were treated with *H. pylori* strains or in combination with verteporfin. Cells were washed three times in cold PBS before adding cell lysis buffer (Cell signaling, Beverly, MA, USA) with protease inhibitor cocktail (Roche, Amherst, CA, USA). Protein concentration was determined using a BCA assays. The cell lysates with an equal amount of protein (25 μg) was separated on 10% SDS-PAGE and transferred to nitrocellulose membranes. The membranes were blocked in Odyssey blocking buffer (Li-COR, Lincoln, NE, USA) at room temperature for 1 h and then incubated with primary antibodies in 5% BSA-TBST at 4 °C overnight. The membranes were then incubated with IRDye-conjugated anti-mouse and anti-rabbit secondary antibodies (1:10000, Li-COR) in Odyssey blocking buffer containing 0.2% Tween-20 at room temperature for 1 h. The protein bands on the nitrocellulose blots were imaged and quantified by the Odyssey imaging system through the analyze module (Li-COR). Band intensity of proteins of interest was normalized to GAPDH.

### Real-time quantitative PCR analysis

For measuring mRNA levels of target genes, total RNA was extracted using Trizol Reagents (Invitrogen) and converted to cDNA using the High Capacity cDNA Archive kit (Thermo Fisher Scientific) according to the manufacturer’s instructions. Levels of *YAP*, *CTGF* and *CYR61* mRNA were measured by qPCR using commercial probe mixtures ((Thermo Fisher Scientific) in the 7500 Fast Real-Time PCR system (Life Technologies). Taqman Fast Universal PCR Master Mix (Thermo Fisher Scientific) was used in this assay. Transcript levels were normalized to the endogenous control glyceraldehyde-3-phosphate dehydrogenase mRNA (*GAPDH*) and expressed as fold change compared with sham-dosed control mice using the Comparative C_T_ method (Applied Biosystems User Bulletin no. 2).

### Cell elongation assay

AGS cells were seeded onto 12-well cell culture plates at a density of 1× 10^5^ cells per well. After incubating for 3~ 4 h, *H. pylori* cells were added at AGS cells at MOI of 200. At 24 h post infection, cells were stained by a three-step staining set (Thermo Fisher Scientific). Images were captured under a contrast microscope (Zeiss Axioskop 2). The length and breadth for at least 30 elongated cells each group were measured using ImageJ software, and then the length-to-breadth ratios were statistically analyzed.

### Immunofluorescence

Cells were washed three times with iced-cold PBS and incubated for 15 min at room temperature with 4% formaldehyde in PBS. The cells were permeabilized with 0.25% Triton X-100 for 15 min and then blocked for 1 h in PBS with 3% bovine serum albumin (BSA). The cells were incubated with primary antibody of YAP or E-cadherin overnight at 4 °C, and then incubation with anti-rabbit-FITC or Alex-Fluor-568-conjugated anti-mouse secondary antibody. Cell nuclei were counter-stained with DAPI. All slides were examined, and images were captured using a fluorescent microscope (Zeiss Axioskop 2).

### Cell migration and invasion assay

For Boyden chamber assay, AGS cells were suspended in 200 μl serum-free DMEM and seeded into a 24-well Boyden chamber (8 μm pore size, Corning, NY, USA) with Matrigel-pre-coated inserts (BD, Franklin Lakes, NJ, USA). The chambers were incubated in DMEM medium with 10% FBS. After a 24 h incubation, cells attached to the chambers’ lower surface were fixed with 4% paraformaldehyde, and then stained with 0.1% crystal violet, and counted under a microscope (Nikon Ti-S). Wound healing assay and transwell assay for cell migration and invasion were performed as previously described [28].

### Gastric specimens and immunohistochemistry

A total of 199 paraffin-embedded human gastric adenocarcinoma and corresponding non-cancerous specimens were obtained from surgical samples without adjuvant therapy. The clinical characteristics of all patients are listed in Additional file 5: Table S2. Additional 230 paraffin-embedded specimens were collected from endoscopic patients diagnosed with chronic non-atrophic gastritis (80 cases), intestinal metaplasia (50 cases), dysplasia (60 cases) and gastric cancers (40 cases). All specimens were provided by The First Affiliated Hospital of Nanchang University. The study protocol and exemption of informed consent were approved by the Ethics Committee of The First Affiliated Hospital of Nanchang University. Status of *H. pylori* infection for these clinical specimens was determined with a rapid urease test and Giemsa staining. Immunohistochemical staining was performed to examine expression profiles of YAP, E-cadherin and YAP/TAZ on these samples as described previously [29], which were evaluated and scored for intensity (scaled 0–3) and frequency (scaled 0–4) by two pathologists blinded to sample identity. For statistical analysis, expression levels of YAP and E-cadherin proteins were illustrated by an expression score in range of 0 to 12 using the formula intensity×frequency [30].

### Statistical analysis

All the statistical analysis was performed using SPSS 20.0 software. Data were presented as mean ± standard deviation (SD) of three independent experiments. Statistical significance of the in vitro studies for continuous variables were determined by one-way Analysis of variance (ANOVA) and Student’s t-test. All comparison protein expression and clinicopathological parameters were performed with Kruskal-Wallis (> 2 groups) or Mann-Whitney tests (2 groups). Pearson correlation analysis was performed for the correlation between YAP and E-cadherin expression. *P* value ≤0.5 was considered significant (***, *P* < 0.001, **, *P* < 0.01, *, *P* < 0.05).

## Results

### Expression of YAP was upregulated in human gastric cancer tissues and was correlated with tumour sizes and metastatic status

**Fig. 1:**
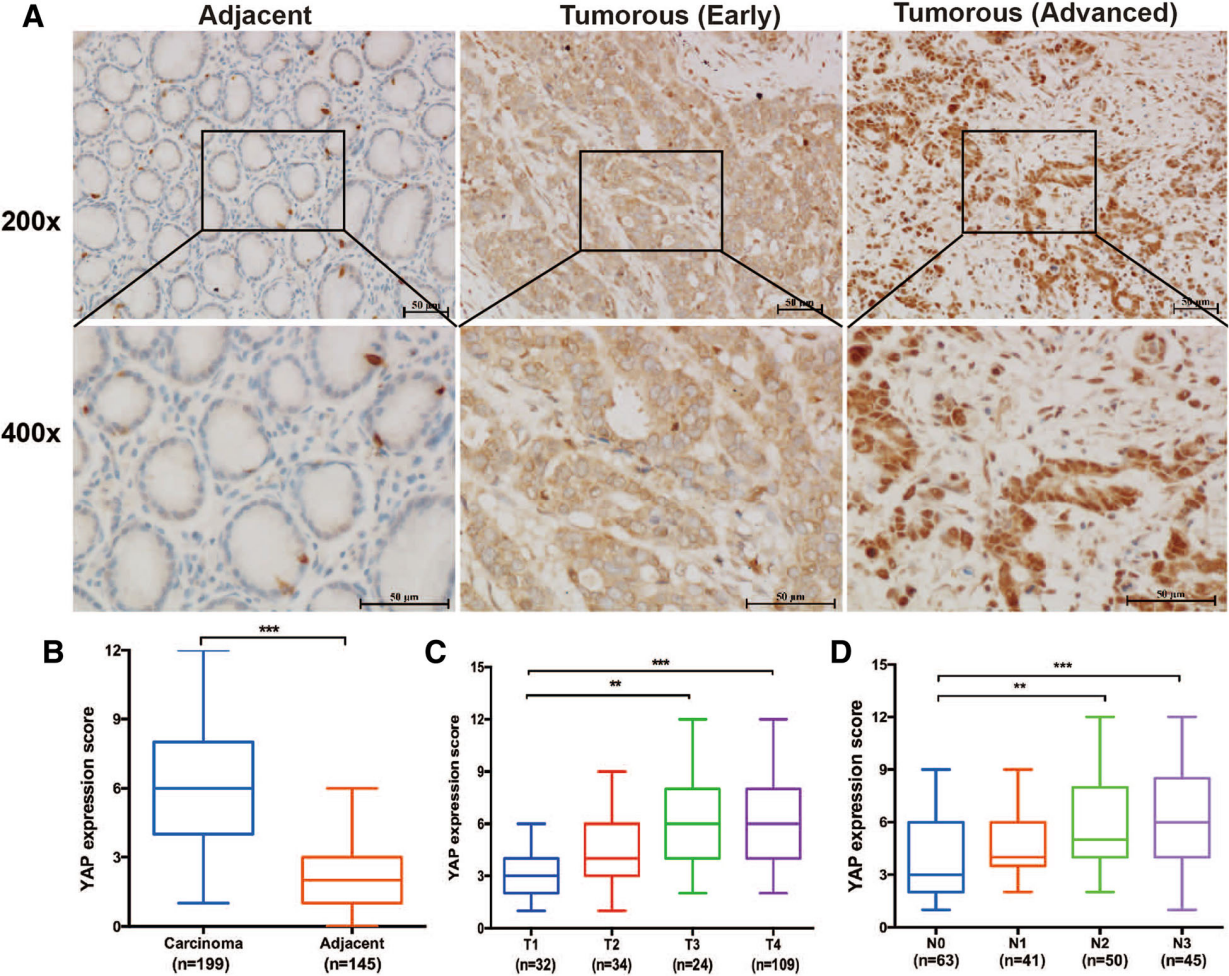
Increased nuclear localization of YAP in human gastric carcinoma tissues compared with their adjacent normal tissues as determined by immunohistochemistry staining. **a** Representative images of YAP protein in adjacent noncancerous tissues, early stage and advanced stage of gastric tumor (Magnification 200×, Scale bars = 50 μm). **b** Quantitative analysis of YAP expression in paired cancer and noncancerous tissues. **c**, **d** YAP immunohistochemical scores at different invasion depth (C) or at different degrees of lymph node metastasis (**d**)

To determine the clinical relevance of YAP expression to the development of human gastric cancer, we assessed and compared expression patterns of YAP using immunohistochemistry in cancerous tissues versus their adjacent normal tissues via tumour resection collected from 199 gastric adenocarcinoma patients. YAP was distributed in both the cytoplasm and nucleus in the majority of gastric tumours (Fig. 1a). Compared with the adjacent noncancerous tissue, expression of YAP was significantly increased in gastric cancer tissues (Fig. 1b). Additionally, higher levels of cytoplasmic YAP were noted in the early tumour stages, whereas YAP was predominantly located in the nucleus at the advanced tumour stages (Fig. 1a, b). We also examined the association of YAP expression with pathologic severity of patients with gastric carcinoma. In addition, the transcriptional co-activator with PDZ-binding motif (TAZ), a paralog of YAP, was also overexpressed in gastric tumours compared with noncancerous tissues (Additional file 1: Figure S1A and B). While YAP and TAZ expression were not associated with gender, age of patients and location (Additional file 4: Table S1 and Additional file 5: Table S2), increased YAP and TAZ levels in the gastric tissues was positively correlated with invasion depth and lymph node metastasis (Fig. 1c, d; Additional file 1: Fig. S1C and D). These data suggested that elevated expression and nuclear translocation of YAP were associated with growth of tumour sizes and metastasis.

### Activation of YAP and suppression of E-cadherin expression was positively correlated with progression of chronic non-atrophic gastritis to gastric cancer and *H. pylori* infection

**Fig. 2:**
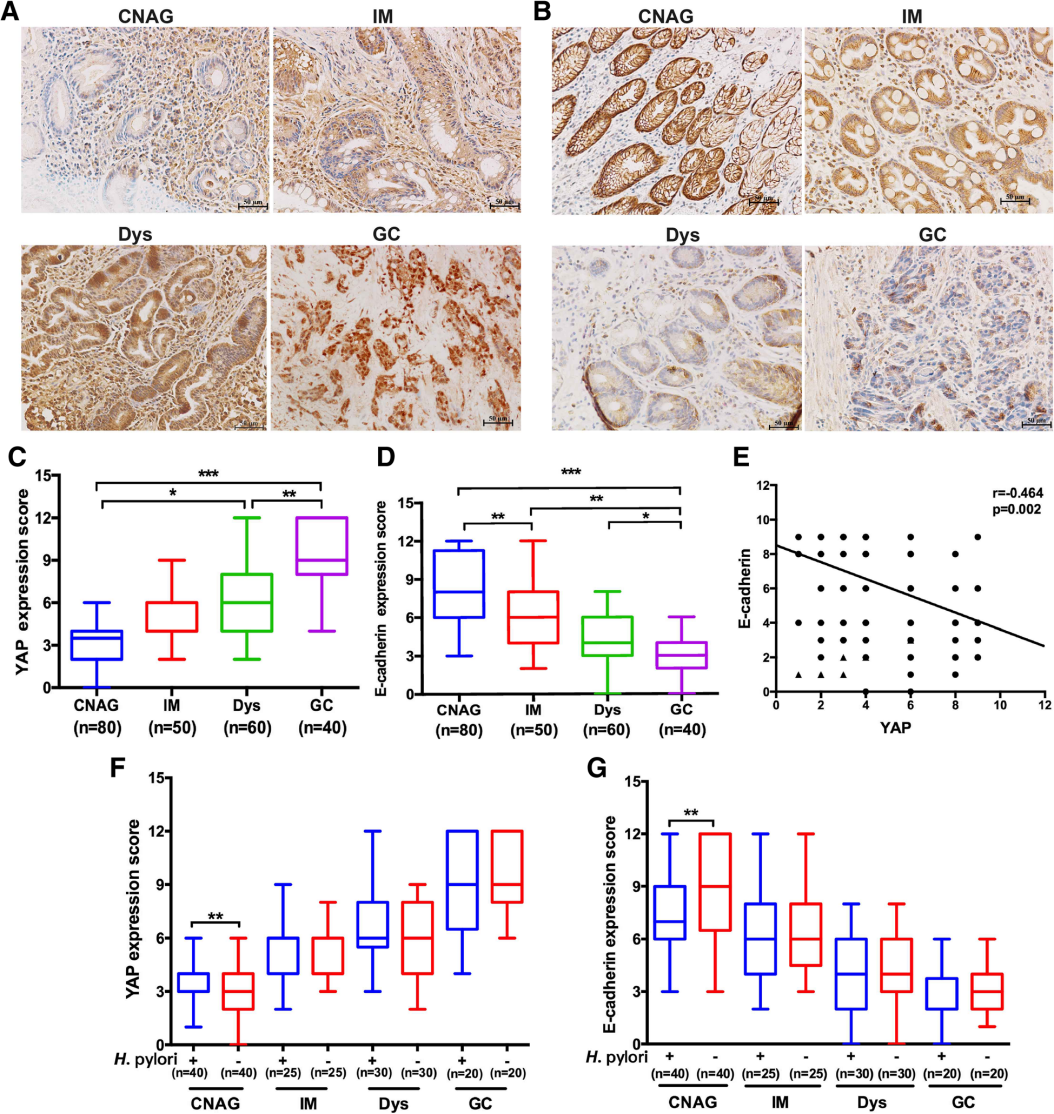
Increase of YAP in concert with a decrease of E-cadherin was positively correlated with progression of the histopathological cascade of human gastric cancer and *H. pylori* infection was associated with higher levels of YAP and lower levels of E-cadherin in the chronic non-atrophic gastritis (CNAG) tissues compared with those in *H. pylori*^−^ CNAG subjects. **a**, **b** Representative images of Immunohistochemical staining for YAP and E-cadherin in human gastric tissues (Magnification 200×, bars =50 μm). Serial tissues were harvested from human gastric mucosa with CNAG, intestinal metaplasia (IM), dysplasia (Dys), gastric carcinoma (GC). Scores were evaluated and statistically compared for expression of YAP (**c**) and E-cadherin (**d**). **e** Pearson correlation indicated that there was a negative correlation between the expression scores of YAP and E-cadherin score (r^2^ = 0.464, *p* < 0.01). Expression levels of YAP (**f**) and E-cadherin (**g**) was compared between *H. pylori*^+^ and *H. pylori*^−^ gastric tissues with different stages (CNAG, IM, Dys, GC) of the Correa histopathological cascade

Progression of chronic non-atrophic gastritis to intestinal metaplasia is a crucial step in the histopathological Correa cascade of gastric tumorigenesis [31]. Additionally, accumulating evidence suggests that activation of the YAP signalling pathway stimulates EMT, a key step for malignant transformation [32]. To explore the potential roles of the YAP signalling pathway and EMT in promoting the progression of gastric tumorigenesis in humans, we examined expression and cellular distribution of YAP and E-cadherin (an epithelial marker for EMT) in 230 human gastric tissues with CNAG, IM, Dys or GC using immunohistochemistry staining. YAP was present in both cytoplasm and nucleus (Fig. 2a), whereas E-cadherin staining was predominantly at the cell membrane (Fig. 2b). Data obtained from quantification of epithelial staining intensity and density indicated YAP levels were gradually increased during neoplastic progression (Fig. 2c), whereas there was a gradual decrease in the expression of E-cadherin (Fig. 2d). Pearson correlation analysis predicted that the levels of YAP expression were negatively correlated with the levels of E-cadherin (Fig. 2e).

Epidemiological data indicate that *H. pylori* is a major risk factor for the development of gastric cancer [33]. To characterize how *H. pylori* infection influenced expression of gastric YAP and E-cadherin, clinical samples were grouped into *H. pylori*^ +^ and *H. pylori*^–^ subjects. We found that *H. pylori*^*+*^ gastric tissues contained significantly higher levels of YAP (Fig. 2f, *P* < 0.01) and lower levels of E-cadherin (Fig. 2g, *P* < 0.01) compared to *H. pylori*^−^ tissues for CNAG but not for IM, Dys and GC (Fig. 2f, g). These results suggest that *H. pylori* infection elevated YAP expression in concert with reduction of E-cadherin in the early stage of the gastric tumorigenesis cascade, which could promote EMT and the possibility of eventual gastric cancer via induction of the YAP signalling pathway.

### CagA^+^*H. pylori* infection elevated YAP expression in gastric epithelial cells

**Fig. 3:**
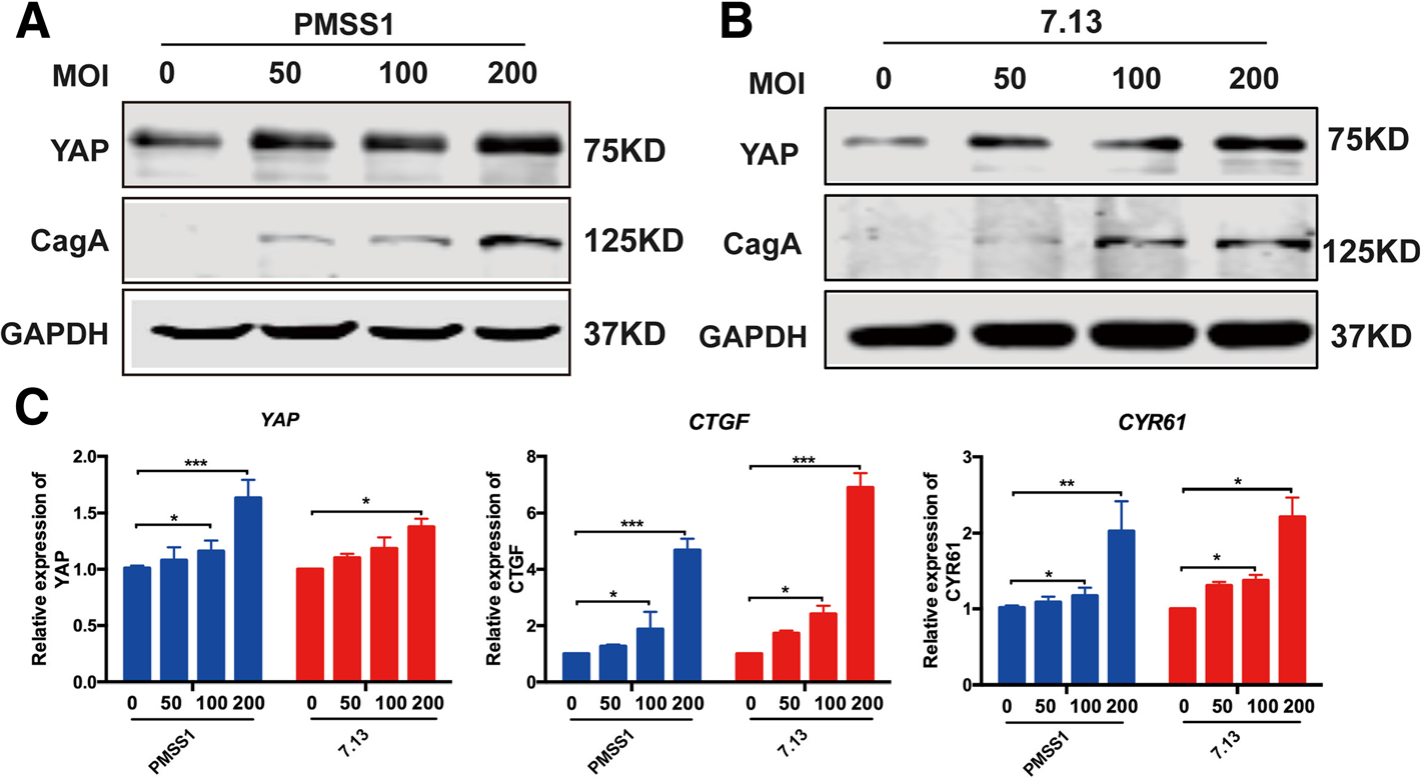
Infection with CagA^+^ but not CagA^−^ *H. pylori* strains activated the YAP pathway signaling. Levels of YAP and CagA in AGS cells which were co-cultured with CagA+ *H. pylori* strains PMSS1 (**a**) or 7.13 (**b**) for 6 h were detected using Western blotting. **c** qPCR quantitation of mRNA levels of *YAP* and its target downstream target genes *CTGF* and *CYR61* was performed

To dissect the possible mechanisms underlying *H. pylori*-associated up-regulation of YAP in clinical subjects with chronic non-atrophic gastritis and characterize a role of CagA, a defined virulence effector of *H. pylori*-induced gastric tumorigenesis, in promoting the YAP pathway, we infected Gastric cell lines AGS with *cagA*^*+*^ and *cagA*^*−*^ *H. pylori* strains. To avoid confusion of CagA status of *H. pylori* strains, CagA^+^ and CagA^−^ are used to represent a given *H. pylori* strain with or without functional CagA throughout the text, respectively. Functional CagA means that this protein can be delivered into and induce cytopathic effects on the host cells. Gastric epithelial AGS cells were co-cultured with CagA^+^ *H. pylori* strains PMSS1 and 7.13 as well as CagA^−^ *H. pylori* strain SS1 at different MOIs (50, 100 and 200) for 6 or 24 h. *H. pylori* SS1, a mouse-adapted strain of PMSS, produces CagA in bacterial cells, but it can’t be delivered into the host cells due to mutations in *cagY* [34]. Infection with strains PMSS1 and 7.13 significantly increased levels of total YAP in AGS cells with a trend in a MOI-dependent manner at 6 h post infection (HPI) (Fig. 3a, b). However, infection with CagA^+^ strains PMSS1 and 7.13 did not affect the ratio of Ser127-phosphorylated YAP to total YAP at 6 HPI (Additional file 2: Fig. S2A and B); this *H. pylori*-induced elevation of YAP was diminished at 24 HPI (Additional file 2: Fig. S2C). In addition, there was MOI-dependent transcriptional increase of *YAP* and its downstream target genes *CTGF* and *CYR61*, most significantly at MOI of 200, in the AGS cells infected with *H. pylori* PMSS1 or 7.13 compared with uninfected controls at 6 HPI (Fig. 3c). In contrast, CagA^−^ *H. pylori* SS1 infection did not significantly enhance YAP levels compared to that in controls at 6 HPI (Additional file 2: Fig. S2D).

### Construction and characterization of PMSS1 isogenic *ΔcagA* mutants

**Fig. 4:**
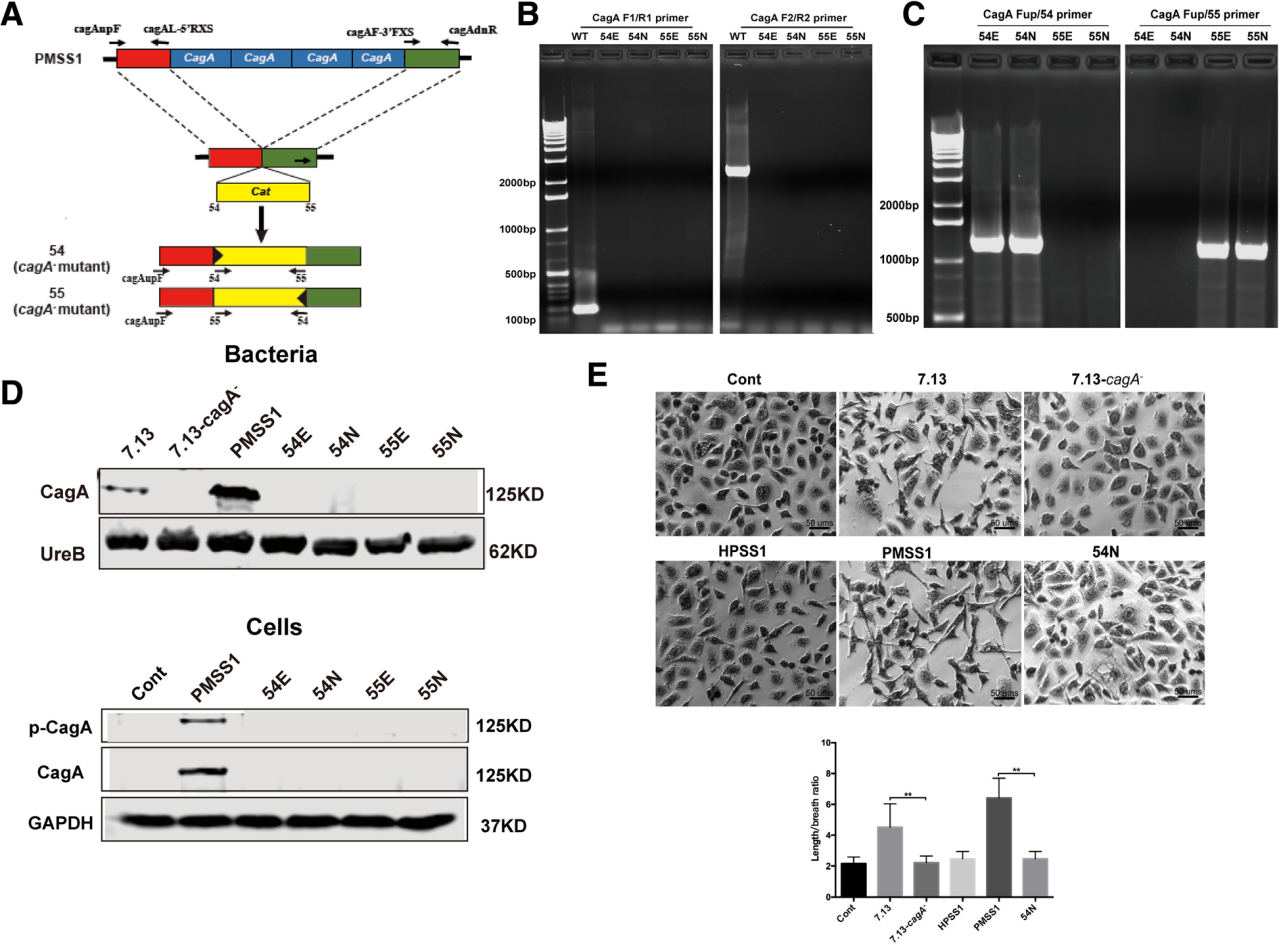
Inactivation of *cagA* decreased expression and nuclear localization of YAP and also downregulated transcription of YAP downstream target genes. Construction and characterization of isogenic PMSS1 *ΔcagA* mutants. **a** schematic depiction of generation of PMSS1 *ΔcagA* mutants. **b**, **c** Complete deletion of *cagA* from the genomes of the representive transformants was completely verified using primer sets F1/R1 and F2/R2 for targeting *cagA* (**b**), and Fup/54 and Fup/55 for targeting the locus containing the sequence upstream of *cagA* and the *cat* cassette (**c**). **d** Western blotting to characterize CagA expression in *H. pylori* cell lysates. UreB was used as a positive control. Total CagA and phosphorylated CagA in AGS cells infected with CagA^+^ *H. pylori* strains PMSS1 and 7.13 as well as their CagA^−^ mutant strains at MOI of 200 for 24 h were detected using Western blotting. **e** Cell elongation assay (Hummingbird phenotype) was performed on AGS cells infected with CagA^+^ *H. pylori* strains PMSS1 and 7.13 as well as their CagA^−^ mutants at MOI of 200 for 24 h. All pictures were obtained under 100× magnification. Degrees of cell elongation (lower right image) was calculated as the ratio of length to breath of a AGS cell

To directly characterize the effect of CagA on the YAP signalling pathway, we constructed isogenic PMSS1 *ΔcagA* mutants. The entire *cagA* region containing variable *cagA* copies in the PMSS1 population was replaced with a chloramphenicol resistance *cat* cassette (Fig. 4a, Additional file 3: Fig. S3A and B) [35]. Isogenic PMSS1 *ΔcagA* mutants were generated by introducing the recombinant plasmids (Additional file 3: Fig. S3C) into recipient PMSS1 cells using electrotransformation or natural transformation, followed by selection on blood agar plates with chloramphenicol. Cm^R^ PMSS1 clones were determined for authenticity of deletion and the *cat* orientation in the PMSS1 genome by PCR assays using specific primers detailed in the Materials and Methods and Additional file 5: Table S2. A PCR amplicon with two specific primer pairs F1/R1 and F2/R2, which targeted the 5′-end and 3′-end regions of *cagA* respectively, was produced from chromosomal DNA templates only from PMSS1, but not from Cm^R^ PMSS1 clones, confirming the complete deletion of *cagA* (Fig. 4b). The transcriptional orientation of *cat*, either opposite or correspondingly to the transcriptional orientation of *cagA* in the mutated *H. pylori* genome, was determined using two primer pairs Fup/54 and Fup/55, respectively (Fig. 4c). Four mutants, designated 54E, 55E, 54 N and 55 N were selected for further characterization. 54 and 55 represented that the transcriptional orientation of *cat,* opposite to *cagA* (54) and along with *cagA* (55) respectively, whereas E and N represented the mutants obtained by electrotransformation (E) or natural transformation (N) (see their identity in Additional file 6: Table S3).

The loss of CagA in the PMSS1 *ΔcagA* mutants was further characterized by examining CagA expression and the ability of these mutants to translocate CagA into AGS cells and cause a cytopathic “hummingbird” phenotype. CagA was present in cell lysates from PMSS1 and 7.13, but was absent from all 4 PMSS1 *ΔcagA* mutants and the 7.13 *cagA-* mutant. In addition, CagA translocation and phosphorylation was detected in the AGS cells co-cultured with parental strains PMSS1 and 7.13 but not with their respective mutants (Fig. 4d and Additional file 3: Fig. S3D). After delivery into gastric epithelial cells, CagA induced the “hummingbird” phenotype [13]. Using this assay, AGS cells infected with CagA^+^*H. pylori* strains PMSS1 and 7.13 exhibited significant cell elongation compared to uninfected controls, whereas the morphology of cells infected with CagA^−^ *H. pylori* mutants, 7.13 *cagA*− or 54 N, was not significantly different from those of the uninfected controls (Fig. 4e). In addition, there was no “hummingbird” phenotype on AGS cell co-cultured with CagA- *H. pylori* SS1. These results collectively demonstrated that PMSS1 *ΔcagA* mutants lost functional CagA.

### *H. pylori* CagA promoted the YAP pathway signalling

**Fig. 5:**
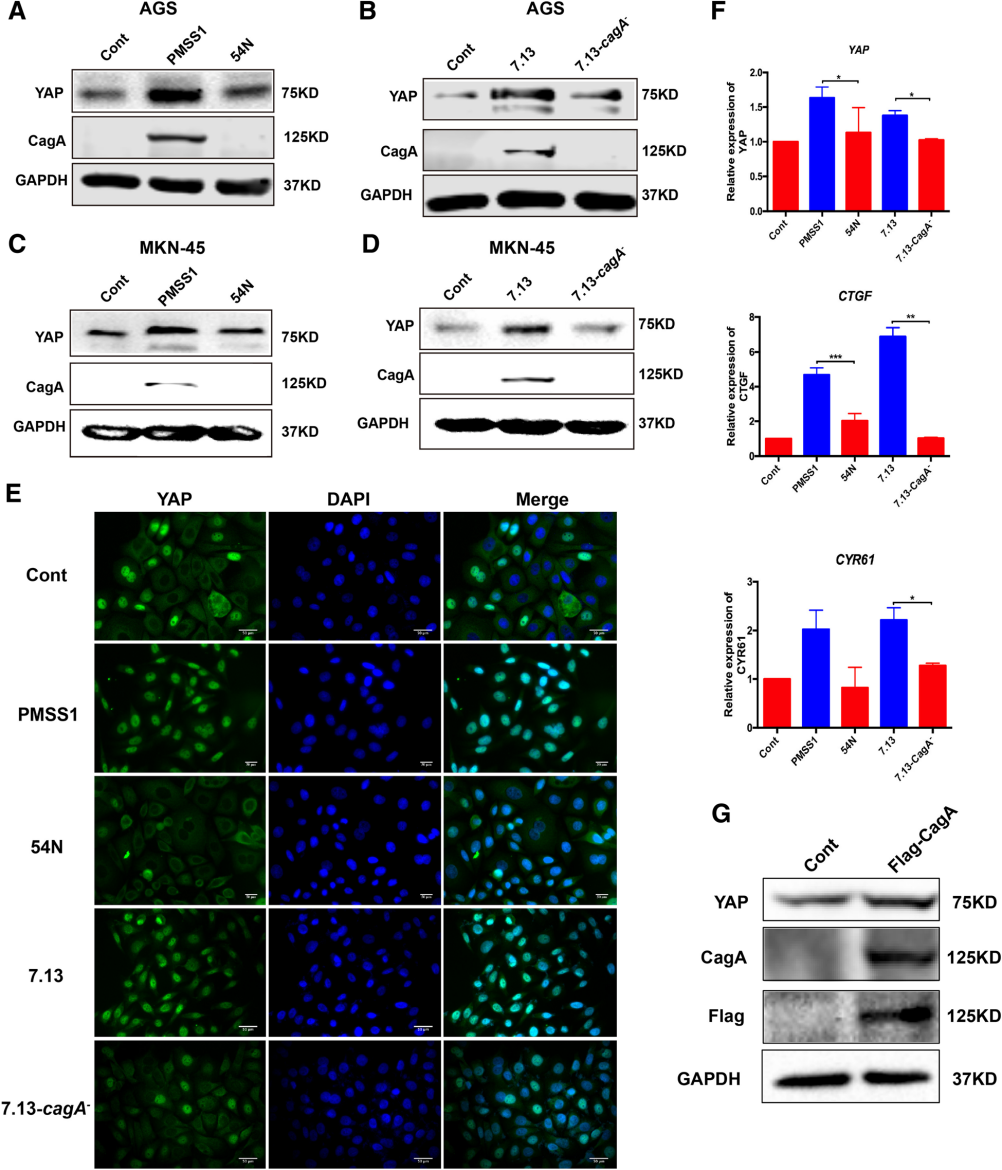
**a**, **b** AGS cells were infected with all *H. pylori* strains at MOI of 200 for 6 h. Levels of YAP and CagA were assessed by Western blotting in AGS cells co-cultured with PMSS1 and its *ΔcagA* mutant 54 N (**a**) or 7.13 and its CagA^−^ mutant (**b**). **c**, **d** YAP and CagA levels were detected in MKN-45 cells. **e** Expression and localization of YAP (green) visualized by immunofluorescence. The blue-fluorescent DAPI was used for nuclear staining. **f** mRNA levels of *YAP* and YAP downstream target genes were assessed by qRT-PCR. Data for gene expression were the mean ± SEM of 3 independent experiments. **g** Representative Western blot for CagA and YAP in AGS cells transfected with the recombinant CagA protein

To investigate the role of CagA in the regulation of the YAP signalling pathway, AGS cells were co-cultured with CagA^+^ *H. pylori* strains 7.13 or PMSS1, and their corresponding CagA^−^ mutants. Levels of *YAP* mRNA and protein in the cells infected with the CagA^−^ strains were comparable to those in the uninfected control cells, whereas infection with PMSS1 and 7.13 significantly increased expression of YAP mRNA and protein when compared with the uninfected controls (Fig. 5a, b and f). Similar effects on YAP expression were obtained from another human gastric cancer cell line MKN-45 co-cultured with CagA^+^ and CagA^−^ *H. pylori* strains (Fig. 5c, d). To further characterize whether elevated YAP enhances its function as a transcription activator, immunofluorescence staining, and qPCR were performed for determining the nuclear localization of YAP and expression of its downstream target genes respectively. Treatment with PMSS1 or 7.13 significantly induced the nuclear translocation of YAP, while infection with *H. pylori* strains lacking CagA resulted in cytoplasmic retention and inactivation of YAP as occurred within the uninfected control cells (Fig. 5e). In addition, levels of *CTGF* and *CYR61* mRNA, whose transcription is controlled by the activation of YAP, were significantly elevated in the AGS cells infected with PMSS1 and 7.13 but not with their CagA^−^ mutants compared to the uninfected controls (Fig. 5f). Furthermore, expression of the recombinant CagA protein vectored by plasmid name (KX673185) in AGS cells significantly elevated protein levels of YAP compared with its empty vector control (Fig. 5g). These data demonstrated that *H. pylori* CagA plays a crucial role in promoting activation of the YAP pathway signalling in this in vitro model.

### CagA^+^*H. pylori* infection enhanced EMT through the activation of the YAP pathway

**Fig. 6:**
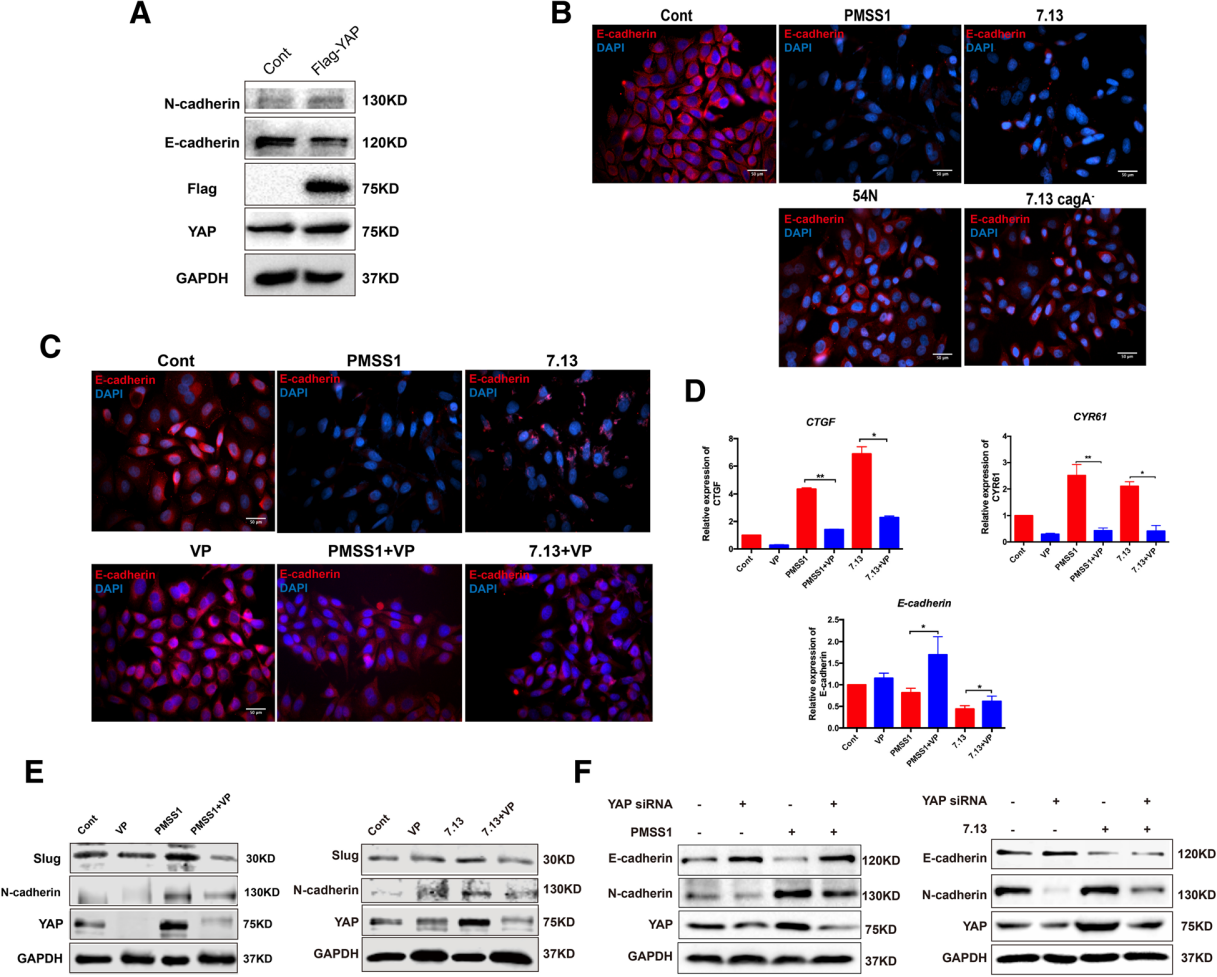
**a** Expression of E-cadherin (an epithelial marker) and N-cadherin (a mesenchymal marker) in AGS cells transfected with YAP cDNA plasmid. **b** After AGS cells were infected with *H. pylori* strains 7.13 or PMSS1 and their *cagA*^*−*^ mutants, Expression of E-cadherin (red) were visualized using Immunofluorescence. **c** Immunofluorescence was performed for E-cadherin levels. **d** mRNA levels of YAP downstream genes *CTGF, CYR61* and E-cadherin (an epithelial marker) in AGS cells infected with *H. pylori* strains alone or in combination with VP treatment. **e** Expression of YAP and mesenchymal markers Slug and N-cadherin in AGS cells treated with CagA^+^*H. pylori* PMSS1 or 7.13 alone or in combination with VP. **f** Expression of YAP, epithelial markers E-cadherin and mesenchymal markers N-cadherin in AGS cells treated with CagA^+^*H. pylori* PMSS1 or 7.13 alone or in combination with YAP siRNA. Data for gene expression are mean ± SEM of 3 independent experiments

Given that the levels of YAP were inversely correlated with the levels of E-cadherin noted in the *H. pylori* ^+^ CNAG tissues vs the *H. pylori*^−^ CNAG tissues and also during the progression of the Correa cascade of human gastric tumorigenesis (Fig. 2c, g), we investigated whether CagA-dependent YAP induction could promote EMT in AGS cells. To evaluate the relationship between YAP expression and EMT, we introduced a plasmid containing the YAP cDNA into AGS cells. Transient overexpression of YAP decreased the level of E-cadherin, an epithelial marker of EMT, indicating that activation of YAP facilitates EMT (Fig. 6a). Treatment with wild-type *H. pylori* strains (7.13 or PMSS1) led to the reduction of E-cadherin expression compared with the uninfected controls as visualized by immunofluorescence, whereas E-cadherin expression were partially restored in the AGS cells infected with CagA^−^ *H. pylori* mutants (Fig. 6b).

To further verify the role of YAP in promoting EMT, we treated AGS cells with verteporfin, a YAP inhibitor through disrupting YAP interaction with TEADs and promoting trypsin cleavage of YAP [36]. YAP expression was decreased by treatment with verteporfin in a concentration-dependent manner (Additional file 3: Fig. S3E). Treatment of verteporfin attenuated partially augmented E-cadherin expression (Fig. 6c) and *H. pylori*-induced mRNA levels of *CTGF* and *CYR61* (Fig. 6d) and in CagA^+^ *H. pylori*-infected AGS cells. In contrast, the levels of Slug (a repressor of E-cadherin expression) and N-cadherin (a mesenchymal marker of EMT) were decreased in the cells treated with both *H. pylori* and verteporfin compared with the cells treated with *H. pylori* alone (Fig. 6e). Furthermore, knockdown of YAP by YAP siRNA in vitro upregulated the epithelial marker E-cadherin which was inhibited by *H. pylori* CagA, and downregulated the mesenchymal marker N-cadherin which was induced by *H. pylori* CagA (Fig. 6f). These findings indicate that activation of YAP played an important role in *H. pylori* infection-induced EMT.

**Fig. 7:**
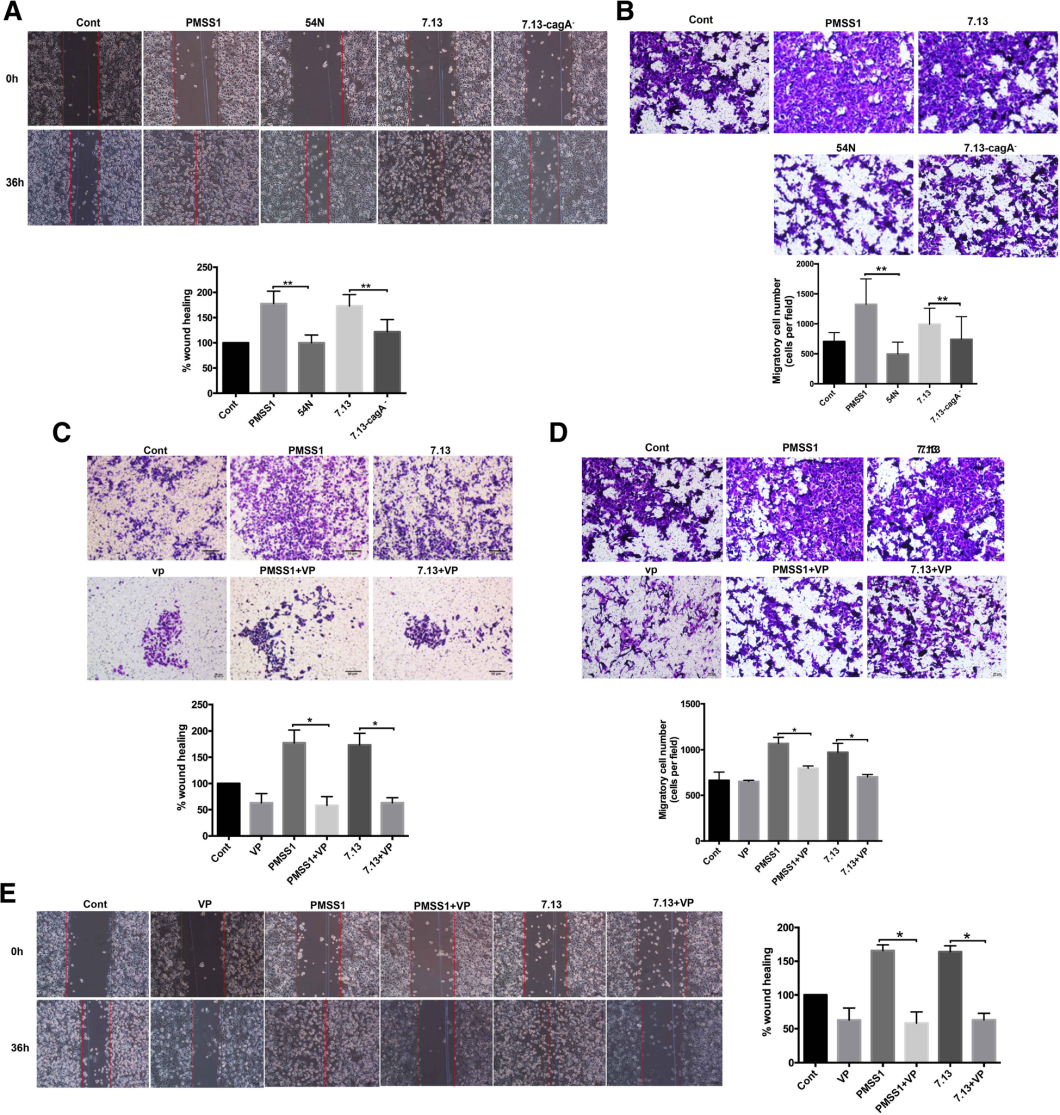
**a**, **b** Wound healing assay (**a**) and Boyden chamber assay (**b**) were performed in AGS cells infected with *H. pylori* wild-type strains (PMSS1 or 7.13) and CagA^−^ mutants. **c** AGS cells were co-cultured with CagA+ *H. pylori* strains PMSS1 or 7.13 in combination with VP treatment, subsequently cells invasion was analyzed by transwell assay. **d**, **e** Cell migration were analyzed by wound healing assay (**d**) and Boyden chamber assay. Data for gene expression are mean ± SEM of 3 independent experiments

Wound healing assays and Boyden chamber assays were also performed to assess the effect of *H. pylori* CagA on the migration and motility of gastric cancer cells. We found that infection with *H. pylori* strains PMSS1 or 7.13 significantly increased the migration of AGS cells, whereas infection with CagA^−^ mutants of these strains inhibited this migratory phenotype (Fig.7a, b). In addition, treatment with a YAP inhibitor verteporfin in combination with *H. pylori* infection significantly suppressed CagA^+^ *H. pylori*-induced gastric cells invasion (Fig. 7c) and cells migration (Fig. 7d, e). Taken together, these findings suggested that activation of YAP promotes *H. pylori* CagA -induced cell invasion and migration.

## Discussion

**Fig. 8:**
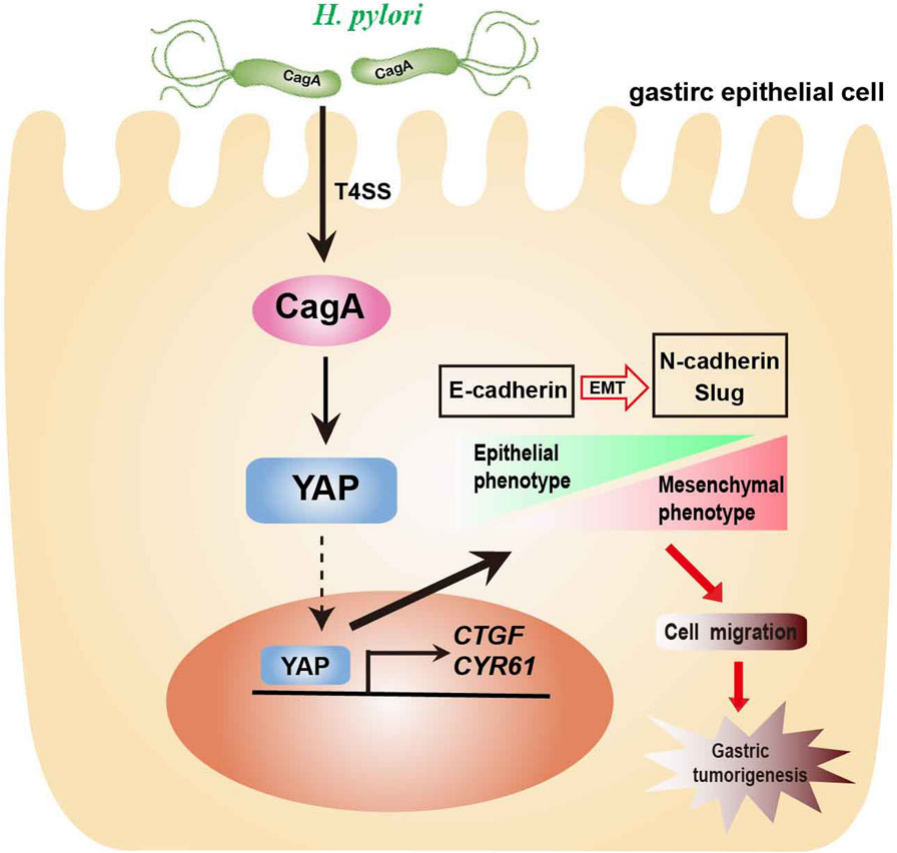
The proposed mechanism of *H. pylori* CagA-mediated promotion of gastric tumorigenesis. CagA^+^ *H. pylori* delivers CagA into gastric epithelial cells via T4SS where CagA induces oncogene YAP expression, increases nuclear translocation of YAP that elevates downstream gene expression. The *H. pylori* CagA-mediated activation of YAP then leads to enhanced EMT program, thereby further promoting gastric tumorigenesis

Epidemiological data indicate that the presence of functional CagA is associated with the higher risk for the development of gastric cancer [37]. It has also been documented in vitro and in vivo that CagA can trigger various pro-oncogenic signaling such as β-catenin, PI3K/Akt, Erk signaling pathways, thereby potentiating the ability of *H. pylori* to induce gastric carcinogenesis [38]. YAP as a key effector of the Hippo signaling pathway is involved in appropriate cellular functionality such as cell proliferation, differentiation, migration and gastric epithelial cells homeostasis. In this study, we showed that there was more YAP expression and stronger the nuclear translocation of YAP in *H. pylori +* CNAG compared with *H. pylori*^−^ CNAG, whereas opposite was true for E-cadherin expression. Subsequently, we demonstrated that CagA functioned as an inducer of the pro-oncogenic YAP signaling pathway in AGS cells, a new mechanism possibly underlying the *H. pylori*-associated elevation of YAP in concert with the decrease of E-cadherin expression noted in human subjects, to promote gastric carcinogenesis. Infection of CagA^+^ *H. pylori* strains PMSS1 and 7.13 as well as recombinant CagA expression led to upregulation of YAP expression, increased nuclear localization of YAP, enhanced expression of downstream genes *CTGF* and *CYR61*, decreased expression of E-cadherin, eventually induced invasion and migration of AGS cells, while these cytopathic effects were suppressed by inactivation of CagA from these *H. pylori* strains or by treatment with verteporfin, a YAP inhibitor or by YAP siRNA. Based on these findings, we propose a working model to illustrate the mechanism underlying the role of CagA in promoting gastric tumorigenesis (Fig. 8). In this model, CagA^+^ *H. pylori* infection further elevates activation of the YAP pathway signaling that enhances EMT (a hallmark of tumorigenic transformation) via skewed expression of mesenchymal factors such as N-cadherin and Slug, thereby promoting cell migration and gastric tumorigenesis.

A previous study showed that there were elevated mRNA levels of YAP and its downstream targets *CTGF*, *CYR61* and *CDX2* in the gastric tissues of C57BL/6 mice treated with a carcinogenic agent MNNG in combination with CagA^−^ *H. pylori* SS1 infection when compared with uninfected controls [26]. This finding appears to be in disagreement with our results that *H. pylori* SS1 and CagA^−^ mutants of *H. pylori* strains PMSS1 and 7.13 did not enhance expression of YAP and its downstream genes *CTGF* and *CYR61* in AGS cells. At least two factors could contribute to this discrepancy. First, experimental systems used in these two studies are fundamentally different. The AGS cell line used in our study were originally derived from a gastric cancer tissue [39]. The direct interaction between AGS cells and *H. pylori* allowed analysis under a more defined condition. In contrast, the responses of C57BL/6 mice to *H. pylori* infection are modulated by a more complicated environment full of cytokines and immune cells involved in pro-inflammatory and anti-inflammatory pathways. Second, in the study of Jiao et al. [26], it is unclear how treatment with MNNG alone or *H. pylori* alone influenced Yap expression in C57BL/6 mice, because these groups were not described in their study.

EMT is frequently activated in cancer invasion and metastasis, and it also contributes to the initiation of gastric adenocarcinoma. During this process, activated EMT reduces gastric epithelial features and confers mesenchymal characteristics [40]. Our results showed that infection with CagA^+^ *H. pylori* strain PMSS1 or 7.13 led to downregulation of the epithelial marker E-cadherin expression in concert with increased expression of a mesenchymal marker N-cadherin, further promoting invasion and migration of gastric epithelial cancer cell lines AGS. Importantly, CagA deficiency in *H. pylori* strains PMSS1 and 7.13 or treatment of verteporfin suppressed YAP expression and partially restored E-cadherin expression and inhibited cell migration. The partial restoration of E-cadherin in the CagA^−^ *H. pylori*-infected AGS cells suggested that additional *H. pylori* factors could also contribute to EMT. Our results are also consistent with a previous study reporting that induction of EMT on AGS cells by *H. pylori* strain 60190 was a CagA-dependent process [25]. Mechanistically, it has been reported that the EPIYA motif of CagA can bind to GSK-3, resulting in depletion of GSK-3 and abnormal expression of various cancer-associated genes including AMPK, β-catenin. Intriguingly, recent study has indicated that YAP enhances the transcriptional activity of β-catenin via GSK-3 activity in glioma progression [41]. Also, the cellular energy sensor AMPK exerts an inhibitory role on the YAP activity via promotion of its phosphorylation [42]. Therefore, these studies suggest that enhanced YAP pathway due to *H. pylori* infection is probably associated with GSK3/β-catenin or AMPK pathway regulated by CagA. Of note, it has been reported that the Hippo signaling pathway suppresses tumorigenesis by phosphorylation of YAP on Ser127, which can restrict YAP to the cytoplasm where phosphorylated YAP undergoes sequestration via binding to 14–3-3 protein. However, in our study CagA^+^*H. pylori* infection elevated the levels of total YAP, but did not alter the ratio of phospho (Ser 127)-YAP to the total YAP. It is likely that the increase of YAP nuclear translocation and upregulation of YAP target genes in the CagA^+^ *H. pylori*-infected AGS cells result from the elevation of unphosphorylated YAP.

It has been proposed that gastric cancer is gradually developed over many years through a multistep histopathological cascade [4]. In this study, we showed that gastric YAP expression also increased, which was correlated with the decrease of E-cadherin, from the non-atrophic gastritis, dysplasia to gastric cancer, further suggesting that the YAP signaling pathway plays a pivotal role in promoting gastric tumorigenesis. More importantly, higher YAP levels and lower E-cadherin levels were noted in the chronic gastritis tissues from *H. pylori*-positive patients compared to those from *H. pylori*-negative patients, suggesting that activation of the YAP signalling pathway at the stage of CNAG is one of the major molecular mechanisms *H. pylori* utilizes to promote the cascade of gastric carcinogenesis. This hypothesis is supported by the results that the promotion effect of CagA^+^ *H. pylori* on YAP expression was diminished at the stages of IM/Dys/GC and also is consistent with our data that enhancement of YAP expression in the CagA^+^*H. pylori*-infected AGS cells occurred at 6 HPI and diminished at 24 HPI. Future investigations into the correlation between *H. pylori* CagA^+^ status and YAP expression in clinical samples will further strengthen the role of CagA in increasing a GC risk via activation of the YAP signaling in the early stage of the development of gastric cancer.

In summary, we have demonstrated that *H. pylori* CagA promotes gastric tumorigenesis via activation of the YAP signalling pathway in AGS cells during the initiation of *H. pylori* infection. These in vitro results are further linked to the clinical observation that *H. pylori* infection elevated activation of the YAP expression in concert with the suppression of E-cadherin in the chronic gastritis tissues of *H. pylori +* patients compared with those of *H. pylori-* patients. These data have highlighted an important role of YAP in CagA virulence potential in particular and gastric tumorigenesis in general. Thus, the findings from this study may have provided a mechanistic insight into why *cagA*^*+*^ *H. pylori* infection is associated with a higher risk for the development of gastric cancer. In addition, further investigations into the correlation among *H. pylori cagA* status, YAP expression and chronic gastritis could potentially develop novel strategies for eradicating *H. pylori* and prevent the development of gastric cancer.

## Conclusions

Our data reveal that *H. pylori* infection activates the YAP signalling pathway to promote EMT in gastric carcinogenesis via CagA, a defined *H. pylori* virulence factor. These findings not only highlight a new mechanistic insight into the role of CagA in promoting *H. pylori*-induced gastric carcinogenesis, but also provide a novel molecular target for developing effective strategies to eradicate *H. pylori* and prevent the development of gastric cancer.

## Additional files

Additional file 1: **Figure S1.** (A) Representative images of Immunohistochemistry staining of TAZ in human gastric carcinoma tissues. (B) Quantitative analysis of YAP expression in paired cancer and noncancerous tissues. (C, D) YAP immunohistochemical scores at different invasion depth (C) or at different degrees of lymph node metastasis (D). (JPG 198 kb)

Additional file 2: **Figure S2.** (A, B) Phosphorylation of YAP was detected using western blotting in AGS cells infected with *H. pylori* 7.13 (A) or PMSS1 (B) strain, respectively for 6 h. (C) Western blotting was performed for YAP expression in AGS cells cocultured with CagA^+^ *H. pylori* PMSS1 strains for 24 h. (D) YAP and CagA were assessed in AGS cells cocultured with CagA^−^ *H. pylori* SS1 strain, at 6 h’ time point. (JPG 204 kb)

Additional file 3: **Figure S3.** Generation and characterization of PMSS1 *ΔcagA* mutants. (A) Overlapping PCR amplicon consisting of the upstream and downstream regions of the PMSS1 *cagA* gene. (B) A 0.7-kb *cat* (chloramphenicol acetyltransferase) cassette digested with *Hinc*II. (C) *Sma*l-digested recombinant plasmid containing the upstream and downstream regions of the PMSS1 *cagA* gene. (D) CagA and phospho-CagA were assessed using Western blotting in AGS cells infected with PMSS1 and its isogenic *ΔcagA* mutants at different MOI for 6 h. (E) Effect of different concentrations of verteporfin (VP) on YAP expression. (JPG 1527 kb)

Additional file 4: **Table S1.** Correlation of YAP expression and clinic pathological status of the patient with GC. (DOCX 14 kb)

Additional file 5: **Table S2.** Correlation of TAZ expression and clinic pathological status of the patient with GC. (DOCX 14 kb)

Additional file 6: **Table S3.** The primers used in this study. (DOCX 13 kb)

## Abbreviations

*cagA*: Cytotoxin-associated gene A
CNAG: Chronic non-atrophic gastritis
CTGF: Connective tissue growth factor
CYR61: Cysteine-rich angiogenic inducer 61
DAPI: 6-diamidino-2-phenylindole
Dys: Dysplasia
EMT: Epithelial-mesenchymal transition
FITC: Fluorescein isothiocyanate
GC: Gastric cancer
*H. pylori*: *Helicobacter pylori*
IM: Intestinal metaplasia
MNNG: Administration of 1-methyl-3-nitro-1-nitrosoguanidine
MOI: Multiplicity of infection
YAP: Yes-Associated Protein
