# Correction: *Helicobacter pylori* CagA promotes epithelial mesenchymal transition in gastric carcinogenesis via triggering oncogenic YAP pathway

**DOI:** 10.1186/s13046-023-02895-8

**Published:** 2023-11-21

**Authors:** Nianshuang Li, Yan Feng, Yi Hu, Cong He, Chuan Xie, Yaobin Ouyang, Stephen C. Artim, Deqiang Huang, Yin Zhu, Zhijun Luo, Zhongming Ge, Nonghua Lu

**Affiliations:** 1 Department of Gastroenterology, The First Affiliated Hospital of Nanchang University, 17 Yong Waizheng Street, Donghu District, Nanchang, 330006 Jiangxi Province China; 2 Division of Comparative Medicine, Massachusetts Institute of Technology, 77 Massachusetts Avenue, Cambridge, MA 02139 USA; 3 Epartment of Biochemistry, Boston University School of Medicine, 72 East Concord Street, Boston, MA 02118 USA

**Correction:** ***J Exp Clin Cancer Res*** **37, 280 (2018)**

**https://doi.org/10.1186/s13046-018-0962-5**

Following the publication of the original article [1], duplicate images were found in Figure 7b and Figure 7d. The correct figure is given below:

**Fig. 7:**
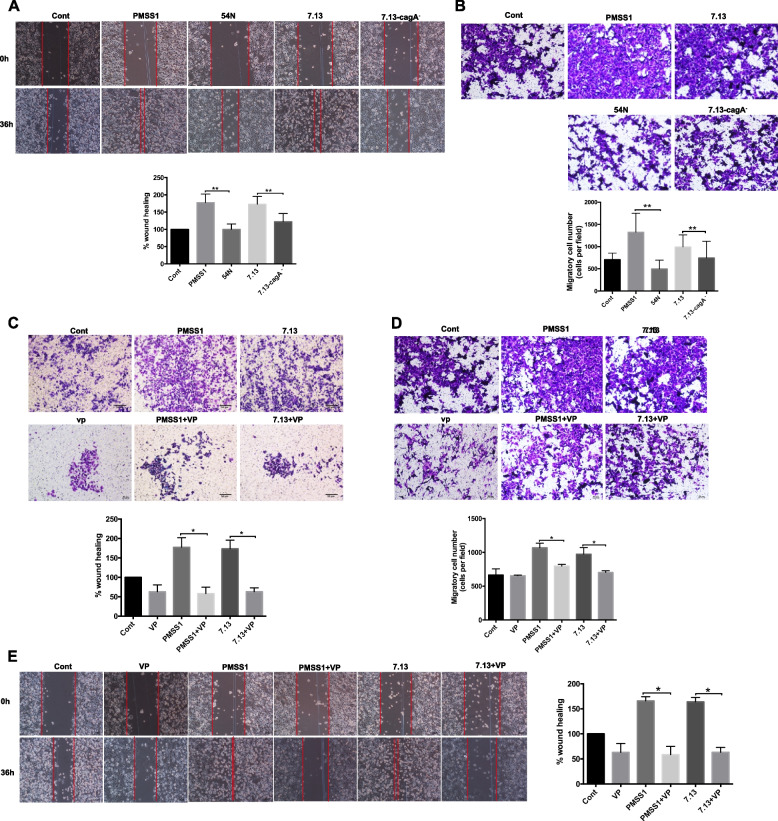
**a, b** Wound healing assay (**a**) and Boyden chamber assay (**b**) were performed in AGS cells infected with *H. pylori* wild-type strains (PMSS1 or 7.13) and CagA − mutants. **c** AGS cells were co-cultured with CagA + *H. pylori* strains PMSS1 or 7.13 in combination with VP treatment, subsequently cells invasion was analyzed by transwell assay. **d, e** Cell migration were analyzed by wound healing assay (**d**) and Boyden chamber assay. Data for gene expression are mean ± SEM of 3 independent experiments
