# Supplementary figures and images for: Helicobacter pylori CagA promotes epithelial mesenchymal transition in gastric carcinogenesis via triggering oncogenic YAP pathway
Source: J Exp Clin Cancer Res. 2018 Nov 22;37:280. doi: 10.1186/s13046-018-0962-5 (PMC6251132; doi:10.1186/s13046-018-0962-5)

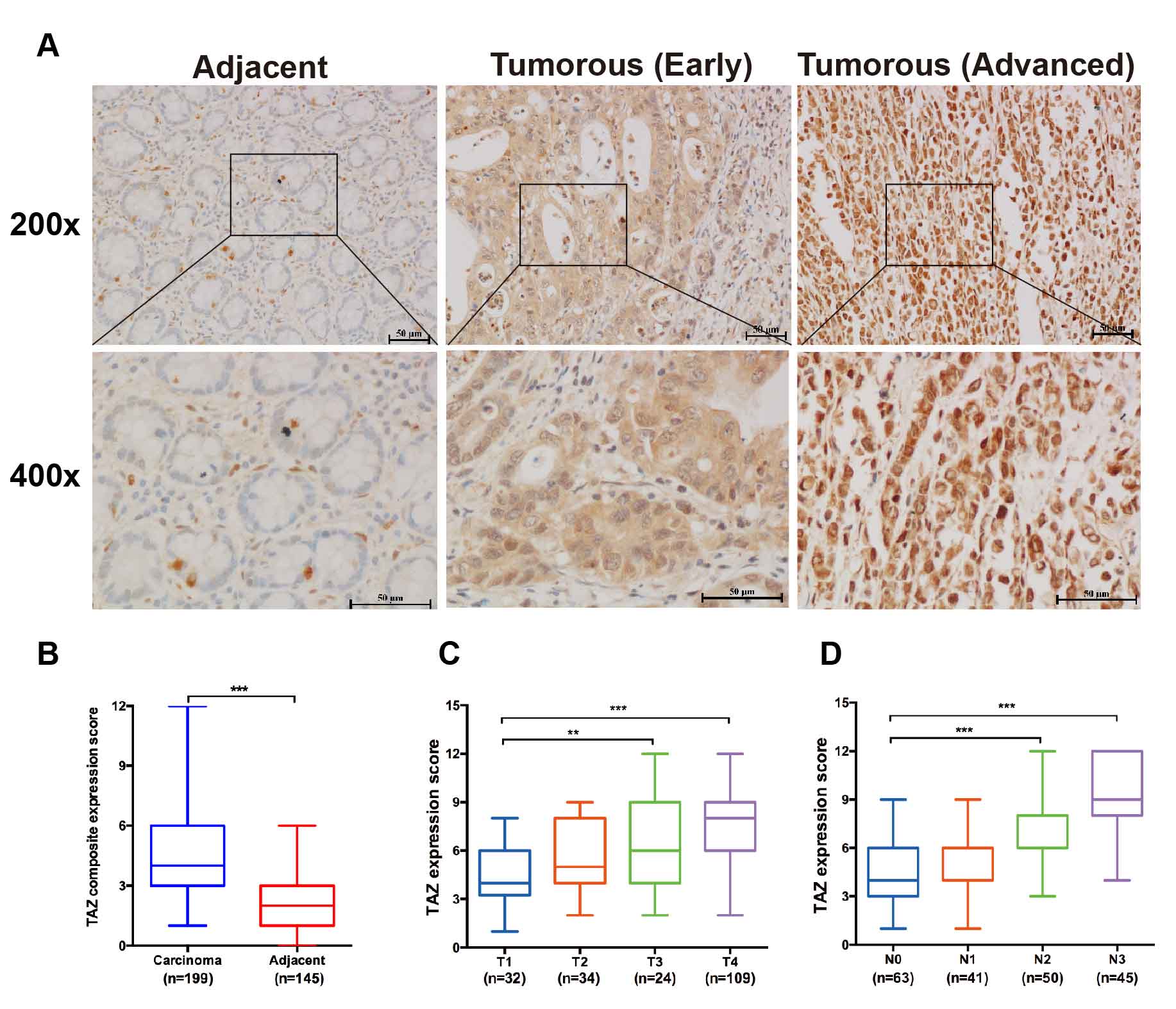

Supplement: Supplementary file 1 — Figure S1. (A) Representative images of Immunohistochemistry staining of TAZ in human gastric carcinoma tissues. (B) Quantitative analysis of YAP expression in paired cancer and noncancerous tissues. (C, D) YAP immunohistochemical scores at different invasion depth (C) or at different degrees of lymph node metastasis (D). (JPG 198 kb) [file 13046_2018_962_MOESM1_ESM.jpg]

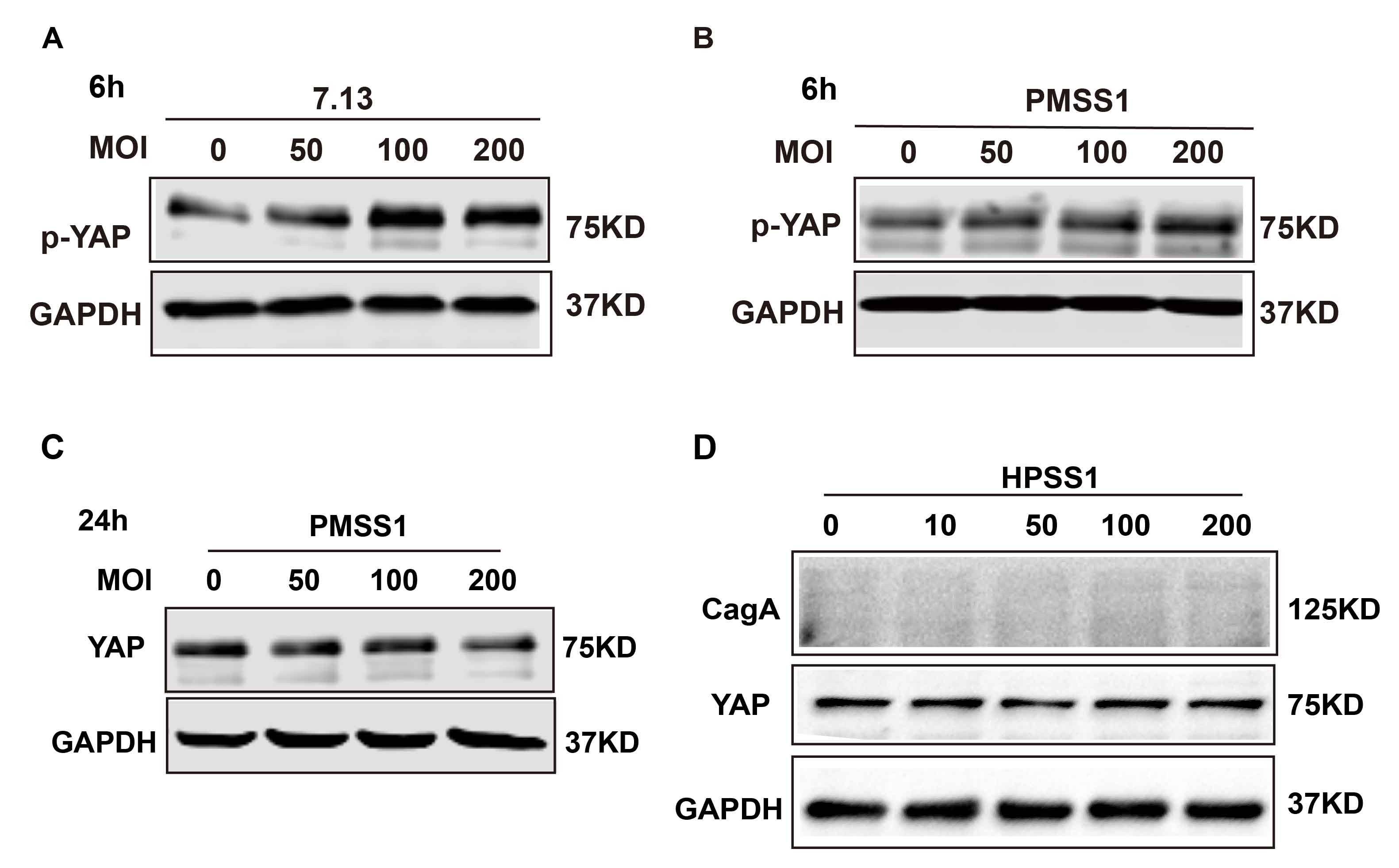

Supplement: Supplementary file 2 — Figure S2. (A, B) Phosphorylation of YAP was detected using western blotting in AGS cells infected with H. pylori 7.13 (A) or PMSS1 (B) strain, respectively for 6 h. (C) Western blotting was performed for YAP expression in AGS cells cocultured with CagA+ H. pylori PMSS1 strains for 24 h. (D) YAP and CagA were assessed in AGS cells cocultured with CagA− H. pylori SS1 strain, at 6 h’ time point. (JPG 204 kb) [file 13046_2018_962_MOESM2_ESM.jpg]

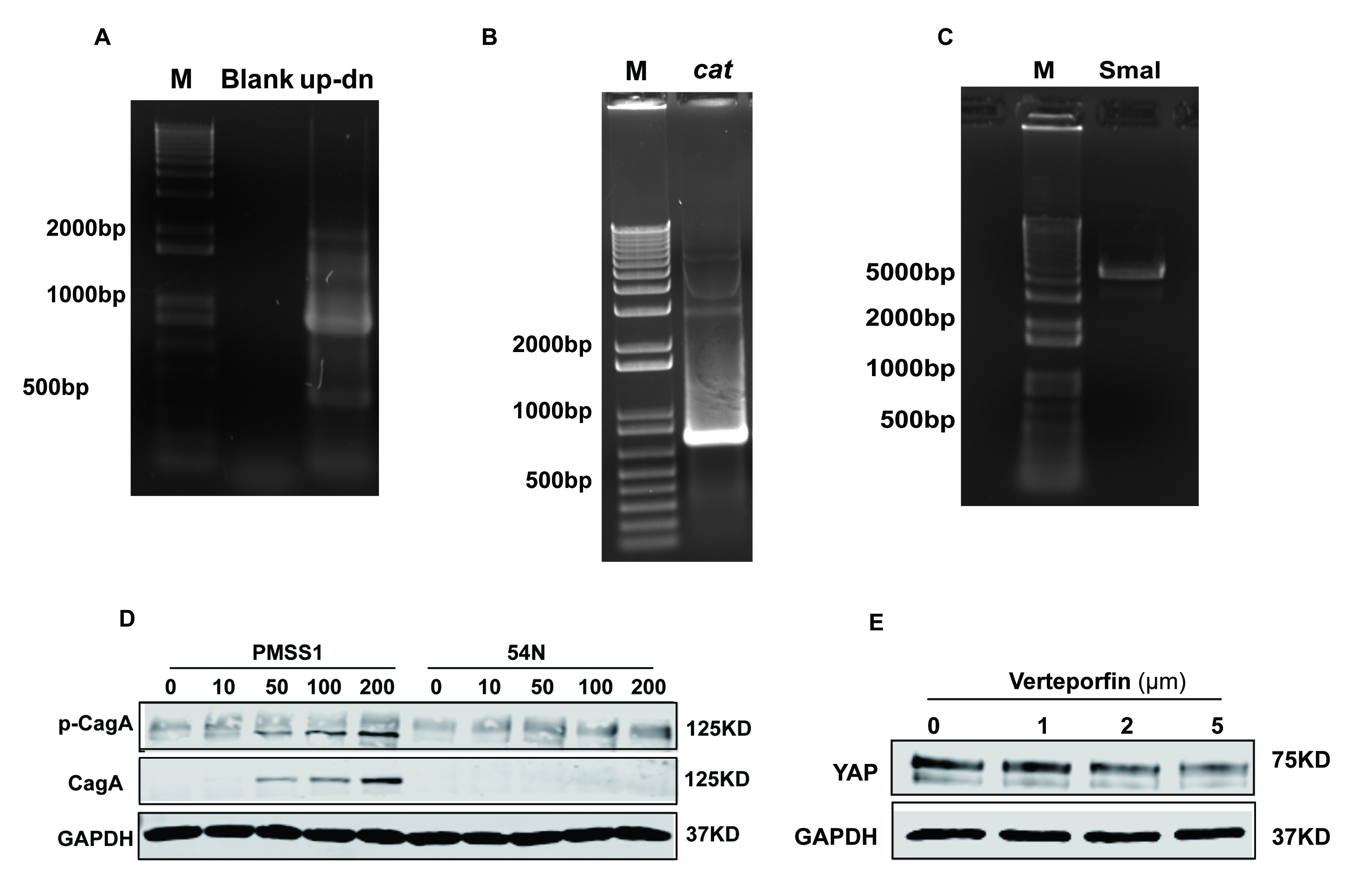

Supplement: Supplementary file 3 — Figure S3. Generation and characterization of PMSS1 ΔcagA mutants. (A) Overlapping PCR amplicon consisting of the upstream and downstream regions of the PMSS1 cagA gene. (B) A 0.7-kb cat (chloramphenicol acetyltransferase) cassette digested with HincII. (C) Smal-digested recombinant plasmid containing the upstream and downstream regions of the PMSS1 cagA gene. (D) CagA and phospho-CagA were assessed using Western blotting in AGS cells infected with PMSS1 and its isogenic ΔcagA mutants at different MOI for 6 h. (E) Effect of different concentrations of verteporfin (VP) on YAP expression. (JPG 1527 kb) [file 13046_2018_962_MOESM3_ESM.jpg]
