# Supplementary material for: Helicobacter pylori CagA promotes epithelial mesenchymal transition in gastric carcinogenesis via triggering oncogenic YAP pathway
Source: J Exp Clin Cancer Res. 2018 Nov 22;37:280. doi: 10.1186/s13046-018-0962-5 (PMC6251132; doi:10.1186/s13046-018-0962-5)
Supplement: Supplementary file 4 — Table S1. Correlation of YAP expression and clinic pathological status of the patient with GC. (DOCX 14 kb) [file 13046_2018_962_MOESM4_ESM.docx]

| **Supplementary Table 1** | | | | | | | |
| --- | --- | --- | --- | --- | --- | --- | --- |
| **The final staining score as indicated were divided into four grades: negative(0~2 ), +(3~5) , ++(6~8) , +++(9~12)** | | | | | | | |
| **Characteristic** | **n** | **YAP expression** | | | | | |
|  |  | -, n | +, n | ++, n | +++, n | %^a^ | p-Value |
| **Age (years)** |  |  |  |  |  |  |  |
| <60 | 92 | 20 | 29 | 34 | 9 | 78.30% | >0.05 |
| ≥60 | 107 | 18 | 46 | 27 | 16 | 83.20% |  |
| **Gender** | | | | | | | |
| Male | 166 | 28 | 59 | 53 | 26 | 83.10% | >0.05 |
| Female | 33 | 11 | 11 | 10 | 1 | 66.70% |  |
| **Location** | | | | | | | |
| Antrum | 109 | 29 | 32 | 32 | 16 | 73.40% | >0.05 |
| Body and cardia | 90 | 12 | 39 | 29 | 10 | 86.70% |  |
| **Tumor Size** | | | | | | | |
| ≤3cm | 70 | 21 | 25 | 17 | 7 | 70.00% | >0.05 |
| >3cm | 129 | 21 | 47 | 43 | 18 | 83.70% |  |
| **Invasion depth** | | | | | | | |
| T1 | 32 | 12 | 14 | 4 | 2 | 62.50% | <0.05 |
| T2 | 34 | 7 | 14 | 10 | 3 | 79.40% |  |
| T3 | 24 | 4 | 6 | 12 | 2 | 83.30% |  |
| T4 | 109 | 15 | 37 | 37 | 20 | 86.20% |  |
| **Lymphonode metastasis** | | | | | | | |
| N0 | 63 | 20 | 26 | 13 | 4 | 68.30% | <0.05 |
| N1 | 41 | 8 | 17 | 12 | 4 | 80.50% |  |
| N2 | 50 | 7 | 19 | 17 | 7 | 86.00% |  |
| N3 | 45 | 6 | 9 | 19 | 11 | 86.70% |  |
| ^a^Percentage of immunostaining. | | | | | | | |
