# Supplementary material for: Helicobacter pylori CagA promotes epithelial mesenchymal transition in gastric carcinogenesis via triggering oncogenic YAP pathway
Source: J Exp Clin Cancer Res. 2018 Nov 22;37:280. doi: 10.1186/s13046-018-0962-5 (PMC6251132; doi:10.1186/s13046-018-0962-5)
Supplement: Supplementary file 5 — Table S2. Correlation of TAZ expression and clinic pathological status of the patient with GC. (DOCX 14 kb) [file 13046_2018_962_MOESM5_ESM.docx]

| **Supplementary Table 2** | | | | | | | | |
| --- | --- | --- | --- | --- | --- | --- | --- | --- |
| The final staining score as indicated were divided into four grades: negative (0~2 ), +(3~5) , ++(6~8) , +++(9~12) | | | | | | | | |
| **Characteristic** | n | **TAZ expression** | | | | | | |
|  |  | -, n | +, n | ++, n | +++, n | %^a^ | p-Value |  |
| **Age(years)** |  |  |  |  |  |  |  |  |
| <60 | 92 | 7 | 26 | 36 | 23 | 92.30% | >0.05 |  |
| ≥60 | 107 | 8 | 28 | 45 | 26 | 92.50% |  |  |
| **Gender** |  |  |  |  |  |  |  |  |
| Male | 166 | 13 | 47 | 68 | 38 | 92.20% | >0.05 |  |
| Female | 33 | 1 | 7 | 14 | 11 | 97.00% |  |  |
| **Location** |  |  |  |  |  |  |  |  |
| Antrum | 109 | 7 | 27 | 43 | 32 | 93.60% | >0.05 |  |
| Body and cardia | 90 | 7 | 27 | 39 | 17 | 92.20% |  |  |
| **Tumor Size** |  |  |  |  |  |  |  |  |
| ≤3cm | 70 | 8 | 24 | 26 | 12 | 88.60% | <0.05 |  |
| >3cm | 129 | 6 | 30 | 56 | 37 | 95.30% |  |  |
| **Invasion depth** |  |  |  |  |  |  |  |  |
| T1 | 32 | 4 | 10 | 14 | 4 | 87.50% | <0.05 |  |
| T2 | 34 | 2 | 14 | 12 | 6 | 94.10% |  |  |
| T3 | 24 | 4 | 8 | 6 | 6 | 83.30% |  |  |
| T4 | 109 | 4 | 22 | 50 | 33 | 96.30% |  |  |
| **Lymphnode metastasis** |  |  |  |  |  |  |  |  |
| N0 | 63 | 10 | 23 | 21 | 9 | 84.10% | <0.001 |  |
| N1 | 41 | 3 | 19 | 17 | 2 | 92.70% |  |  |
| N2 | 50 | 1 | 8 | 30 | 11 | 98.00% |  |  |
| N3 | 45 | 0 | 4 | 14 | 27 | 100.00% |  |  |
| ^a^Percentage of immunostaining. | | | | | | | | |
