# Supplementary material for: Helicobacter pylori CagA promotes epithelial mesenchymal transition in gastric carcinogenesis via triggering oncogenic YAP pathway
Source: J Exp Clin Cancer Res. 2018 Nov 22;37:280. doi: 10.1186/s13046-018-0962-5 (PMC6251132; doi:10.1186/s13046-018-0962-5)
Supplement: Supplementary file 6 — Table S3. The primers used in this study. (DOCX 13 kb) [file 13046_2018_962_MOESM6_ESM.docx]

|  |  |
| --- | --- |
| **Supplementary Table 3 Primers used in this study** | |
| **primer name** | **Sequence (5'→3')** |
| cagAupF | ACG CCT AAG TTT GGA ATC TGG |
| cagAdnR | GGT TGC ACG CAT TTT CCC |
| cagAF-3'FXS | CTC GAG CCT CCC GGG TTC ATA AAT CTC TCT AAA GCG |
| cagAL-5'RXS | CCC GGG AGG CTC GAG TCT TAG GAT CGT AAA ATT GCG |
| cagAF1 | GGA GCC AAG CAC GAT TGG AAC G |
| cagAR1 | CTT GAC TCA ATG CTC GTT GTG A |
| cagAF2 | CCC CTA TCC CTG ATG ACA A |
| cagAR2 | TGA GAT TGT CTA TCG TTT GTT CTA GAT |
| cagAFup | ATG ACT AAC GAA ACT ATT GAT CA |
| 54 | ATA TGT CGA CGT GAT ATA GAT TGA AAA |
| 55 | TAT AGT CGA CAG TGC GAC AAA CTG |
